# Phenotyping the Prediabetic Population—A Closer Look at Intermediate Glucose Status and Cardiovascular Disease

**DOI:** 10.3390/ijms22136864

**Published:** 2021-06-25

**Authors:** Elena Barbu, Mihaela-Roxana Popescu, Andreea-Catarina Popescu, Serban-Mihai Balanescu

**Affiliations:** Department of Cardiology, Elias Emergency University Hospital, Carol Davila University of Medicine and Pharmacy, 011461 Bucharest, Romania; elena.lechea@drd.umfcd.ro (E.B.); serban.balanescu@umfcd.ro (S.-M.B.)

**Keywords:** atherosclerosis, ASCVD, macrovascular disease, insulin resistance, prognosis, prediabetes cluster

## Abstract

Even though the new thresholds for defining prediabetes have been around for more than ten years, there is still controversy surrounding the precise characterization of this intermediate glucose metabolism status. The risk of developing diabetes and macro and microvascular disease linked to prediabetes is well known. Still, the prediabetic population is far from being homogenous, and phenotyping it into less heterogeneous groups might prove useful for long-term risk assessment, follow-up, and primary prevention. Unfortunately, the current definition of prediabetes is quite rigid and disregards the underlying pathophysiologic mechanisms and their potential metabolic progression towards overt disease. In addition, prediabetes is commonly associated with a cluster of risk factors that worsen the prognosis. These risk factors all revolve around a common denominator: inflammation. This review focuses on identifying the population that needs to be screened for prediabetes and the already declared prediabetic patients who are at a higher risk of cardiovascular disease and require closer monitoring.

## 1. Introduction

Even though the term prediabetes has been around for more than ten years, the medical community is still divided into believers and non-believers. The term prediabetes characterizes a state in which glucose levels do not meet the diagnosis criteria for diabetes but are above the accepted “normal” range.

Isolated impaired glucose tolerance (IGT), isolated impaired fasting glucose (IFG), and IGT or IFG combined with high HbA1c define several forms of prediabetes [1,2].

A recent article analyzed the accuracy of the term prediabetes [3]. In fact, this issue has been continuously debated in the past 10 years and it reflects the lack of international consensus [4]. As defined by the American Diabetes Association ADA, the condition affects more than 84 million US citizens and more than 1 billion people worldwide, creating a huge socio-economical and psychological impact. One-third of Americans older than 18 years meet the criteria for prediabetes, according to the Centers for Disease Control (CDC) [3]. Other organizations including the WHO, the UK National Institute for Health and Care Excellence, the European Association for the Study of Diabetes (EASD), and the International Diabetes Federation (IDF) are more cautious in using the term prediabetes as it is defined by the ADA, especially when using the HbA1c criteria.

There is also a risk that people will presume that all prediabetes patients will eventually develop diabetes while all other patients will not. These assumptions should be avoided, as neither of the two hypotheses has been thoroughly demonstrated [3].

It is easy to understand why US health organizations use the term prediabetes more freely than the European ones, given the epidemic of obesity in the US population, with the related insulin resistance (IR), secondary glycemic disorders, and their consequences. However, the epidemic is turning global [5].

Consequently, it is time to reconsider the importance of intermediate glycemia states and some important arising issues. Is prediabetes harmful on its own or due to the progression to diabetes? What are the characteristics that help distinguish prediabetes patients who develop type 2 diabetes mellitus (T2DM) from those who do not? Are there other clinical and metabolic aspects that should be considered other than high blood sugar? The straightforward answer could be dyslipidemia, hypertension, sedentary lifestyle, obesity, smoking, and heredity factors—but which are the most ill-fated associations?

It is worth mentioning that the risk of developing diabetes does not only depend on the intermediate glycemia status alone but is also dependent on age, sex, body mass index, and genetic, environmental, or ethnic characteristics, thus leading to great variability in the measurement of progression from prediabetes to diabetes, depending on the inclusion criteria of the studied population. It has also been demonstrated that within the intermediate hyperglycemia range, higher values are associated with a greater rate of progression towards diabetes [1].

Therefore, although the cut-offs and the name are still highly debated, the condition is of interest considering its prevalence, the associated risks of developing diabetes and cardiovascular disease, and the burden it imprints on patients and society. The current review focuses on what factors carry a worse prognosis when associated with prediabetes and whether a sub-phenotyping strategy would benefit high-risk patients in terms of early intervention and prevention of cardiovascular disease development.

## 2. Definitions of Prediabetes—What Is the Appropriate Definition?

The diagnosis of prediabetes is established by fasting plasma glucose (FPG), 2 h plasma glucose (PG) during 75 g oral glucose tolerance test (OGTT), or the level of glycated hemoglobin (HbA1c) [6,7]. The issue of diverse definitions and criteria for diagnosing prediabetes makes it difficult to compare and sum up findings from different clinical studies (see Table 1). Studies still show inconsistent results regarding the 100–125 mg/dL FPG cut-off impact on cardiovascular and all-cause mortality [8,9,10,11]. Furthermore, the most recent ESC guidelines on diabetes, prediabetes, and cardiovascular disease take into consideration both recommendations [12]. However, it seems that the ADA criteria are the most used in clinical trials [13].

There is another important aspect when discussing the accuracy of a prediabetes definition. A single HbA1c measurement is 49% sensitive and 79% specific for prediabetes, while FPG has a mean sensitivity of 25% and specificity of 94% [14]. HbA1c alone identified 14% of individuals diagnosed with IGT, 9% with IFG, and 33% with both abnormalities [15]. Additionally, hemoglobin variants, genetic hemoglobinopathies, thalassemia, and iron deficiency should be considered when using HbA1c for intermediate hyperglycemia states diagnosis [6]. This underscores the need to be more cautious and validate the diagnosis with another blood glucose measurement before initiating treatment [16,17].

When establishing the HbA1c cut-off for the diagnosis of prediabetes, expert committees had to rely on information about the shape of risk curves for complications such as retinopathy in the absence of clinical trials that determine the exact moment when the intervention results in the delay of diabetes and its complications [18]. A systematic review demonstrated that a value of HbA1c > 6.0% is associated with a risk of developing T2DM from 25% to 50%, and a value of Hb A1c between 5.5% and 6.0% is linked to a five-year diabetes incidence of 9% to 25% [16,17].

A recent study found that the definition of prediabetes matters, in the sense that the HbA1c cut-off recommended by ADA was superior in discriminating between patients at risk of adverse cardiovascular events and death [19]. In addition, the prediabetic patients defined by FPG or IFG have a better prognosis than patients defined by HbA1c. However, it was the accumulation of risk factors that increased the risk of prediabetic patients, not the prediabetic status itself [20]. Another meta-analysis looked at employing WHO and ADA definitions in studies on middle-aged individuals. The reported prevalence of prediabetes was only 27% when overlapping both sets of criteria and 49% for the ADA criteria alone, while 71% of the latter were identified using the HbA1c criteria. Interestingly, only 8.7% and 3.9% of these prediabetic patients showed a combination of IGT, IFG, and high HbA1c with the ADA criteria and with the combined criteria, respectively. This analysis proves once again that the underlying pathophysiological mechanisms are different, as demonstrated by the limited overlap of patients identified through different criteria [14]. Thus, phenotyping this population is warranted [21].

On the matter of progression to diabetes, a recent meta-analysis demonstrated that the definitions of prediabetes currently in use have a similar ability to predict the risk of conversion to diabetes within 5 years, but did not analyze the risk of Major Adverse Cardiovascular Events (MACE) [22].

## 3. A Modifiable, but Often Overlooked Risk Factor?

Epidemiologic studies place prediabetes as a strong predictor of cardiovascular disease (CVD) [23,24]. However, the good news is that diet, exercise, and weight loss can prevent T2DM. This has already been demonstrated in multiple studies, some of them conducted by the Diabetes Prevention Program Research Group [25,26].

Patients with acute coronary syndromes (ACS), without a previous history of diabetes, and with high fasting blood glucose levels are known to be at increased risk. Whether this is a consequence of the acute stress reaction or a marker of prediabetes and true disturbance of the glucose metabolism is established afterward, in the post-acute phase [27]. The class I recommendation to assess glucose homeostasis in all ACS patients prior to discharge emphasizes just that [28]. Currently, the effects are more visible in primary rather than in secondary prevention of macrovascular complications and mortality [29]. This makes prediabetes a modifiable risk factor that should not be overlooked [30].

In a meta-analysis of 53 prospective cohort studies involving over 1.6 million participants, Huang et al. found that, after correcting for other risk factors, prediabetes was associated with increased risk of all-cause and cardiovascular mortality. However, this could be observed only when defining prediabetes as FPG > 110 mg/dL, IGT, or combined FPG > 110 mg/dL and/or IGT alone. For FPG of 100–125 mg/dL, the risk was greater only in young and middle-aged males, according to subgroup analyses [11].

## 4. Underlying Pathophysiology Mechanisms in Prediabetes

It is well established that T2DM is a heterogeneous disease revolving around beta-cell failure. Several studies concluded that pathophysiological heterogeneity exists before the diagnosis of diabetes, although this fact is not yet reflected in the current definition of prediabetes [31]. While isolated IGT is mostly associated with obesity and IR, the primary abnormality in isolated IFG is β-cell dysfunction with impaired insulin secretion [1]. These distinct conditions, which can present with or without overlap, are included in the definition of prediabetes, and are actually considered as the same disorder, although they may have different associations with clinical events and mortality, and although prevention programs work differently depending on the main pathological mechanism.

There are two main hypotheses for the early stages of intermediate glucose states. The first one focuses on the beta-cell dysfunctionality and considers it the central abnormality. The hostile environment due to inherited predisposition and metabolic stress provokes beta-cell overstimulation which leads to secondary hyperinsulinemia. Basal hyperinsulinemia promotes IR through multiple mechanisms. Insulin excess is associated with lipogenesis and obesity, leading to IR. The disproportionate tissue response to high levels of circulating insulin is also considered as an adaptive response that protects against hypoglycemia. Ultimately, in this scenario, the beta-cell fails [32]. This course of events shows a greater association with IFG rather than IGT and it characterizes predominantly younger people, without obesity or inadequate fat distribution [33,34].

The other hypothesis is that hyperinsulinemia is a compensatory response to tissue decrease in insulin sensitivity. Multiple factors and their interaction are to blame for increased IR, such as excessive food and additives, saturated fatty acids, body fat composition, adipokines (adiponectin, resistin, leptin) and hepatokines (fetuin-A), alterations in bile acid metabolism, gastro-intestinal tract nutrient sensing, and intestinal microbiomes and chronic inflammation sustained by obesity. Eventually, beta-cell exhaustion occurs, leading to a reduction in insulin secretion and progression to T2DM [32,35,36]. IR is generally linked to obese individuals with IGT [34]. In clinical research, IR is estimated using the Homeostasis Model Assessment of IR (HOMA-IR). The cut-off value for HOMA-IR that identifies individuals at risk of developing T2DM appears to vary between different populations, with values ranging from 1.7 to 3.87 [37]. Some studies attempt to establish a cut-off value between prediabetics and diabetics, with prediabetic HOMA-IR values ranging from 1.4 to 3.63 [38,39].

Although prediabetes is managed nowadays as a unitary condition, prediabetic patients express these two mechanisms heterogeneously. Progression to T2DM occurs due to a combination of decreased insulin tissue cell sensitivity and loss of compensatory insulin secretion. IR and beta-cell failure co-evolve in various proportions simultaneously rather than sequentially [40].

In the past 30 years, numerous studies have concluded that IFG and IGT have different underlying pathological mechanisms and different outcomes [41]. IFG and IGT overlap only partially and the addition of these two intermediate glucose states doubles the risk of developing T2DM [42]. Even if both IFG and IGT are characterized by IR, the tissue with inadequate insulin response is different. IFG is associated predominantly with hepatic IR and normal muscle insulin sensitivity. People with IGT mainly have muscle IR [43,44,45]. The timing of abnormal insulin secretion is different for IFG and IGT. IFG shows a reduced early-phase (first 30 min) insulin response to OGTT and a normal late-phase (60–120 min). The IGT state is associated with both early phase and late phase insulin secretion deficit [45].

Abnormalities preceding diabetes are expressed in different proportions, dividing the prediabetic population into several categories or sub-phenotypes [36]. This raises the assumption that, if different pathways in the development of T2DM are considered, there will be differences in the natural evolution and the response to therapeutic strategies.

Dividing patients with prediabetes into distinct categories may be strenuous, considering the different pathways found at many levels before the development of T2DM, but beneficial, considering the burden of the global epidemic.

## 5. Associations with Other Risk Factors: A Cluster of Bad Omen

An essential problem is that most prediabetic patients do not only have glycemic disorders, they also have other cardiovascular risk factors. This emphasizes the need for early identification of prediabetic patients and primary prevention of atherosclerotic cardiovascular disease (ASCVD). The components of metabolic syndrome are often found in prediabetic patients, long before the transition to T2DM. The average prevalence of metabolic syndrome in industrialized countries is 31%. The metabolic syndrome doubles the risk of ASCVD and is accountable for increasing the risk of progression to diabetes four to five times. The risk of all-cause mortality is also multiplied 1.5 times [46].

**Figure 1:**
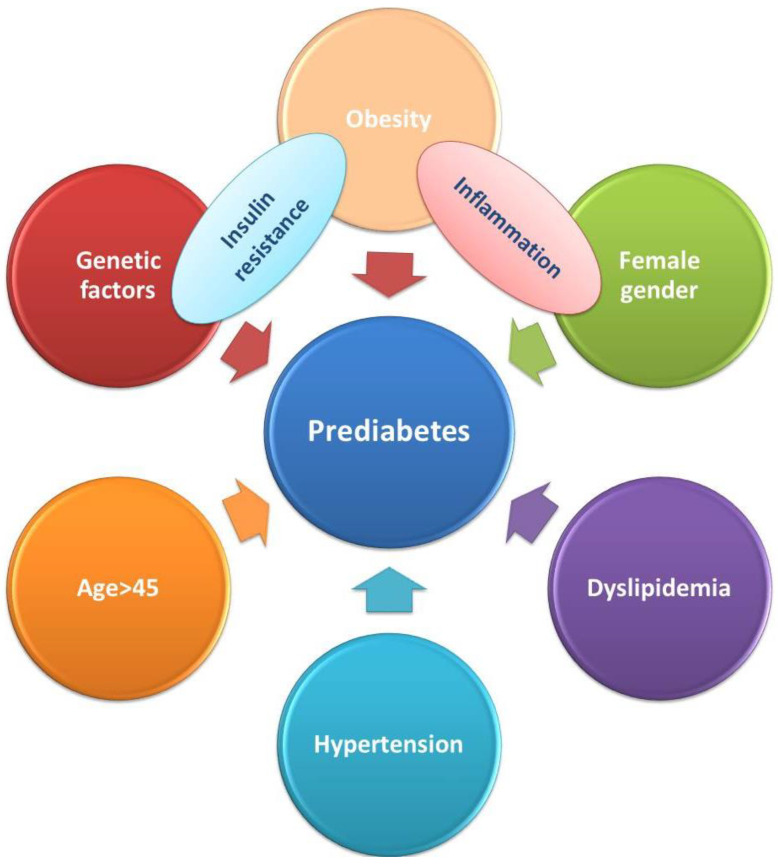
Risk factors associated with prediabetes.

Furthermore, mechanisms other than hyperglycemia that coexist and are interdependent are involved in the onset and progression of diabetes and its complications, especially cardiovascular disease. Therefore, adding to the hyperglycemia environment, increased secretion of adipocyte cytokines (adipokines) and oxidative stress with secondary chronic inflammation that promotes vascular endothelial dysfunction, hypertension, atherogenic dyslipidemia and a prothrombotic status also contribute to the development of CVD, even in the absence of progression to full-blown diabetes (see Figure 1).

### 5.1. Obesity

Obesity counts for about 80–85% of the overall risk of developing diabetes [47]. Whole-body magnetic resonance imaging and spectroscopy used to assess fat distribution in the human body identified distinct phenotypes of obesity according to their metabolic relevance. Therefore, obese people were classified into metabolically healthy and metabolically unhealthy [36]. Upper body obesity, especially visceral obesity, is associated with IR, while obese people with predominantly lower body fat storage and subcutaneous storage are included in the insulin-sensitive phenotype. Furthermore, it seems like lower body obesity is not associated with increased cardiovascular risk [48,49,50]. The amount of liver fat is the main determinant of IR, and phenotypes of benign obesity are characterized by reduced ectopic fat in the liver [36,50].

The cause of different outcomes resulting from the location of fat has not been yet totally explained. Different theories are taken into consideration. One of them is that a common factor produces both main features of metabolically unhealthy obesity: central obesity and IR. According to this theory, IR is not a consequence of visceral obesity. Interestingly, fat storage has a major impact on IR, even in people with normal weight. It seems that decreased insulin sensitivity is associated with a severe dysfunctionality of adipose tissue found in lipodystrophic diabetes. This condition is mainly considered to be a consequence of genetic abnormalities, involved in both lipodystrophy and IR, while other publications blame the deficiency of secretory products of adipocytes as the cause of decreased insulin sensitivity [51,52].

Alternatively, other theories consider that some biochemical characteristics of intra-abdominal fat trigger IR [51]. Visceral obesity is implicated in the development of T2DM through increased free fatty acid flux and increased triglycerides storage in other organs implicated in glucose metabolism, with secondary disturbance in insulin sensitivity, hyperinsulinemia, and beta-cell failure. Moreover, the secretory function of adipocytes has an important role in the development of T2DM and its complications. It seems that fat tissue secretes TNF alpha, plasminogen-activator inhibitor-1, angiotensinogen, leptin, resistin, adiponectin, and other hormones including estrogen and cortisol [51]. Cytokine secretion and hormonal imbalance lead to chronic inflammation that aggravates the insufficient tissue response to insulin, endothelial dysfunction, and atherogenesis, and promotes micro and macrovascular complications.

Again, understanding the existence of distinct phenotypes of fat distribution and function in people with blood sugar abnormalities facilitates prevention interventions that trigger the main pathological pathway, which, at least in theory, may stop or delay the course of prediabetes.

### 5.2. Dyslipidemia

Dyslipidemia and hyperglycemia are traditional modifiable risk factors for cardiovascular disease. There is a two-way connection between lipid and glycemia disorders. Each of these conditions exacerbates the effects of the other, and the two of them combine to create the proper environment for the appearance and progression of diabetes and its complications. Dyslipidemia is considered a traditional risk factor for prediabetes because it can increase IR. Thus, lipid profile may identify prediabetic individuals at high risk for diabetes and CVD complications [53].

Atherogenic dyslipidemia, characterized by increased total cholesterol, small dense low-density lipoprotein (LDL), triglyceride (TG), very low-density lipoprotein (VLDL), TG/HDL ratio and LDL/HDL, and decreased high-density lipoprotein (HDL), is frequently found not only in diabetic patients but also in individuals with intermediate state hyperglycemia compared with normoglycemic patients [54,55]. High LDL is associated with an increased probability of prediabetes, after proper adjustments for age and body-mass index (BMI), regardless of the definition used [56]. The incidence of ASCVD (coronary heart disease or stroke) is higher in prediabetic patients when pooled with elevated lipoprotein (a) levels, as compared to normoglycemic patients [57].

Therefore, on the one hand, IR and hyperinsulinemia can cause lipid disorders, predominantly the phenotypes that lead to ASCVD. On the other hand, the addition of the hyperglycemic environment in a patient with dyslipidemia worsens both conditions and leads to progression to diabetes and CVD.

Subclinical atherosclerosis, the fundamental initial lesion that leads to ASCVD, is mainly found in patients with dyslipidemia and prediabetic status combined, suggesting that, at least for the cardiovascular complications, the prevention interventions should be applied to this category of patients. When considering the distinct prediabetes categories (diagnosed by FPG or HBA1c), both combined with dyslipidemia lead to structural vessel damage compared to normoglycemic and normolipidemic controls. There was a higher association with subclinical atherosclerosis in the presence of a combination of HbA1c and dyslipidemia rather than FPG and dyslipidemia. The risk increased if sub-phenotypes were combined [58].

Other investigators reported that prediabetes displays an increased risk of subclinical atheromatous disease only in the presence of three or more other cardiovascular risk factors such as dyslipidemia, hypertension, and obesity [59]. This aggregation is vastly studied as metabolic syndrome and is associated with an increased, well-established risk of cardiovascular mortality and morbidity.

### 5.3. Hypertension

Hypertension is independently associated with cardiovascular risk. The addition of hypertension to a hyperglycemic environment worsens the prognosis, as discussed above. Whether hypertension is a risk factor for developing diabetes is less clear at this moment. As far as pathological mechanisms go, the hemodynamic changes with secondary endothelial dysfunction and inflammation that accompany hypertension are implicated in the development and progression to T2DM [60]. Additionally, higher angiotensin II activity in hypertensive patients may lead to islet fibrosis via AT1R stimulation, with consequently decreased insulin secretion [61].

Among the 30 cohort studies that investigated the connection of blood pressure (BP) and diabetes, there was a variable strength of association between these two conditions, and 12 of them did not find any association at all. A 20 mmHg elevation of systolic BP and 10 mmHg higher diastolic BP were associated with more than 50% increased risk of diabetes, but the strength of correlation between hypertension and diabetes onset declined when adjusted for age, obesity and sex [62]. Therefore, it is clear that the combination of risk factors in different proportions is the determinant of diabetes progression and complications.

In a recent study, prediabetes was not associated with an increased CVD risk when compared to normoglycemic individuals. However, when glycemic disorders were associated with hypertension, the risk for more severe coronary lesions was significantly elevated [60].

### 5.4. Ethnicity

Ethnicity is a determinant of prediabetes prevalence. The risk of developing diabetes is higher among the African American, Hispanic, and Asian populations [63]. They are also diagnosed at a younger age, and their prognosis is also worse due to the development of complications compared to the Caucasian population [64,65]. Statistical analyses applied on a cohort of almost 5 million individuals from different US regions revealed that ethnicity was an important factor in the progression of diabetes. Ethnic minorities had a higher prevalence than the white population at lower BMIs, suggesting that there are specific racial factors that determine a higher risk of prediabetes and diabetes [66]. The levels of HbA1c may have ethnic variations, independent of the glycemic levels even in the absence of hemoglobin variants [67]. Adjusted cut-offs or the addition of other tests may prove useful in such groups.

### 5.5. Gender

There is a gender difference in the risk factors associated with prediabetes regarding the progression to diabetes [68]. Obesity, alcohol consumption, smoking, and dyslipidemia are more efficient as risk predictors in male patients, while hypertension and poor dietary habits are prominent in women [68].

Prediabetes in women, who are already more prone to inflammation than men, leads to accelerated endothelial dysfunction (preferentially coronary microvascular dysfunction as opposed to obstructive coronary disease) [69]. Indeed, diabetic women have a higher risk of vascular death than men, not explained by the traditional risk factors [70]. In addition, polycystic ovary syndrome (PCOS) is a condition accompanied by insulin resistance, obesity, and low-grade chronic inflammation. The disease quadruples the risk of developing T2DM. The age of diabetes diagnosis is also lower in women with PCOS [71]. The cardiovascular risk, especially for hypertension, is also higher for this category of women [72].

Gestational diabetes is considered a risk factor for diabetes and testing every three years is recommended [67]. However, only one in ten patients with gestational diabetes benefit from follow-up, even though they are at a high risk of developing diabetes later in life [73].

Sudden cardiac death appears to be higher in women with DM and, contrary to what is valid for the general population, the female gender is not protected against premature ASCVD if they are diabetic [12]. The excess relative risk of vascular events in diabetic patients is greater in women and at younger ages, thus research into prediabetes is warranted for this subgroup [12].

### 5.6. Smoking

Smoking is associated with inflammation, thus aiding in the progression from normal to hyperglycemia. It also seems to reduce the peripheral uptake of glucose, leading to IR. Both active and passive smoking accompany intermediate glucose status and diabetes [61].

### 5.7. Inflammation

Inflammation is the common denominator of all these risk factors, the initiator or the aggravator in the cascade of events pertaining to atherosclerosis, obesity, diabetes, dyslipidemia, or hypertension. Its fundamental role, although well established in all of these conditions, is still a matter of substantial research nowadays, in a continuous attempt to establish the exact mechanisms and their role in pathology and clinical outcomes. Although inflammation is present in every single one of them, with distinct or common pathways, neither prediabetes, diabetes, hypertension, nor obesity benefit at this moment from a specifically designed anti-inflammatory treatment or prevention thereof.

## 6. The Endgame: Cardiovascular Morbidity and Mortality

### 6.1. Atherosclerotic Cardiovascular Disease (ASCVD)

In patients with coronary artery disease, the prevalence of intermediate glucose states ranges between 19% and 36% [29]. For peripheral artery disease, the prevalence of prediabetes varies between 26% and 28% [74,75,76]. The mechanism behind this interrelation is associated with vascular inflammation and early endothelial dysfunction. These processes generate a subintimal accumulation of monocytes and T lymphocytes and fatty streak formation. Subsequently, atherosclerotic plaque progression to complicated plaques leads to acute cardiovascular events, aggravated by the fact that hyperglycemia in itself promotes hypercoagulability [77]. Moreover, new experimental data suggest that transient intermittent hyperglycemia can accelerate the atherosclerotic process through myelopoiesis and monocyte production [78]. Even mild or moderate increases in glycemic levels seem to bring about the development of ASCVD [79,80].

It is still not clear which parameter best indicates the presence of ASCVD. Some studies find a better correlation for HbA1c with ASCVD than for IFC or IGT [81]. Some older studies identify only a modest correlation between IFG and vascular disease [82]. It was recently reported that IFG alone was not linked to cardiovascular events, but when associated with hypertension or dyslipidemia the risk was increased [83]. A meta-analysis looking into 53 prospective cohort studies found an increased risk of CVD in individuals with IFG > 100 mg/mL (5.6 mmol/L) or HbA1c of 5.7% (39 mmol/mol), fitting the ADA criteria [84]. However, a recent, updated analysis including 129 studies reports that prediabetes is associated with a higher risk for CVD and all-cause mortality, regardless of the diagnostic criteria used [85].

Another means to estimate the risk of cardiovascular events in prediabetic patients is through IR. Increased IR in non-diabetic patients has been associated with vulnerable atherosclerotic plaques in both stable angina and acute coronary syndrome undergoing percutaneous coronary intervention (PCI) [86,87]. Non-diabetic patients with an increased HOMA-IR had a higher risk of MACE after an acute coronary syndrome and higher rates of echolucent plaques, as identified through intravascular ultrasound (IVUS) [88].

Prediabetes induces subclinical myocardial damage, assessed by higher levels of high-sensitivity cardiac troponin T (hs-cTnT ≥ 14 ng/L) and subsequent clinical events, especially heart failure and mortality [89]. Moreover, patients identified as prediabetics using the HbA1c criteria rather than IFG showed a higher risk of subclinical myocardial damage that might be attributed to microvascular disease [89]. A later study by the same research group identified a strong association between elevated hs-cTnT and the risk of incident diabetes during a 13-year follow-up in patients without known CVD. Interestingly, this correlation was stronger for patients without traditional risk factors. Elevated hs-cTnT was associated with both CVD and diabetes risk, raising the question of a possible overlap in pathophysiological mechanisms of both diseases [90].

However, screening and revascularization of silent CAD are not recommended in diabetics, as it fails to significantly reduce cardiac adverse events [91]. Thus, primary prevention seems to be the key approach in prediabetic patients.

For patients with already established coronary artery disease, after coronary artery bypass grafting (CABG), all-cause mortality and cardiovascular hospitalization were greater in prediabetic patients than in normoglycemic patients [92].

Hospitalization for cardiovascular as well as for endocrine, respiratory, gastrointestinal, neoplasm, genitourinary, neurologic, and infectious causes was 1.3 times higher in prediabetics identified through an HbA1c > 5.7% than in normoglycemic patients [93].

A meta-analysis showed a progressively higher risk of cardiovascular events along the glycemic spectrum, as prediabetes (diagnosed with IFG and/or IGT) exhibited an increased risk as compared to normoglycemia [94]. This trend was confirmed by a recent meta-analysis, with a higher risk of MACE and death in the undiagnosed prediabetic population suffering an AMI [95]. Furthermore, in a large, multiethnic cohort that comprised 44% prediabetic patients, a 20 mg/dL fasting glucose increase was associated with a 17% increment in the risk of MACE [96].

As previously stated, the prevalence of prediabetes among patients with ASCVD might be associated with the presence of other risk factors, which must be taken into account as confounders when calculating the intrinsic risk of hyperglycemia alone of producing ASCVD [82]. In addition, further studies need to be undertaken to estimate the predictive value of HbA1c, as opposed to IFG or IGT.

Although there is a multitude of studies looking into the associations between prediabetes, nephropathy, retinopathy, and neuropathy, this connection is less well defined than the one for macrovascular disease [97].

### 6.2. Heart Failure

IR and hyperglycemia exert deleterious effects on the structure of the heart in the absence of ASCVD or hypertension. They contribute to ventricular fibrosis and hypertrophy, followed by chamber stiffness and diastolic dysfunction [29,98]. Even early left ventricular dysfunction was associated with prediabetes, along with right and left ventricular diastolic impairment [99].

In the context of IR and diastolic dysfunction, it appears that diabetic women have a 5 times higher risk as compared to men (2.4 times higher risk) to develop heart failure [69].

As many as 40% of the patients with heart failure have prediabetes [100,101,102,103]. The CHARM study found that a 1% increase in HbA1c levels leads to a 25% higher risk of a cardiovascular event in patients with symptomatic heart failure [104]. Other studies reported a connection between prediabetes at admission for heart failure and major adverse cardiovascular events [100,101,103].

### 6.3. Acute ASCVD and Cardiovascular Mortality

In a 19-year follow-up study, IFG was shown to increase the risk of sudden cardiac death (SCD), with a 1.51 times higher relative risk of SCD compared to normoglycemic patients [8]. In a cohort of postmenopausal women, IFG was associated with CVD risk and all-cause mortality [105]. However, another study adjudicates the superiority of IGT over IFG to predict all-cause mortality and cardiovascular disease [106].

In older patients (66–90 years old), over a 5-year follow-up period, only long-standing diabetes had an impact on survival. Prediabetes showed no correlation with all-cause and cardiovascular mortality in this subgroup of patients [107]. However, there was evidence of arterial disease demonstrated by increased arterial stiffness (higher brachial-ankle pulse wave velocity (baPWV)) in prediabetics defined by both IFG and HbA1c criteria [108]. Nevertheless, in the same cohort, prediabetic patients reverted to normal glucose levels or died rather than progressing to diabetes. This raises the question of whether the prediabetic status really should be of great concern in older age [109].

The prevalence of prediabetes is increased in the population presenting with acute myocardial infarction (AMI) [27,110]. AMI is accompanied by increased glycemia, without previously diagnosed diabetes, that was originally attributed to acute stress. This supposition was contradicted by the fact that HbA1c at admission and FBG at discharge are independent predictors of glucose intolerance at 3 months post-discharge [27,110]. These parameters can be used in order to identify high-risk individuals for the long-term prognosis. Moreover, stress (admission) hyperglycemia in non-diabetics identifies patients at risk of in-hospital mortality, reflecting a more profound glucose metabolism disturbance [111,112]. Other reports suggest that the long-term prognosis is better estimated using the OGTT [113,114].

## 7. Who Should Be Screened for Prediabetes? Risk Scores

What patient characteristics make them susceptible to prediabetes? There is a continual search for risk-scores and models able to predict which patients are at high risk for incident diabetes and the subsequent worse prognosis. The factors usually taken into consideration are gestational diabetes, diabetic first-degree relative, metabolic syndrome, etc. [115]. The current ADA recommendations for prediabetes screening place obesity and age above 45 in the center of attention. Therefore, overweight or obese adults with one or more risk factors for diabetes should be tested for prediabetes (see Table 2). If there are no additional risk factors, consider screening individuals no later than when they are 45 years old. Screening for glucose abnormalities is also recommended in pregnant women at 24–28 weeks gestation [67].

European and American diabetes associations developed online risk scores for diabetes, that use similar criteria: age, waist, weight, ethnicity, hypertension, family history of diabetes, and physical activity (https://riskscore.diabetes.org.uk/start// accessed on 15 March 2021; https://www.diabetes.org/risk-test// accessed on 15 March 2021, FINDRISC Diabetes Risk Calculator, QxMD).

Multiple other measurements, mostly anthropometric, have been studied for predictive purposes in prediabetes. Raised waist to height ratio > 0.5 was independently associated with a high risk of prediabetes in both genders [116]. The triglyceride glucose (TyG) index (TyG index = Ln[(triglyceride(mg/dL) × glucose(mg/dL)/2]) was also best correlated with prediabetes in both sexes [117,118] in comparison with obesity indices of BMI and waist, lipid profiles of TG, and HDL-C, TG/HDL-C, and was superior to FPG in female and obese patients [119]. In other studies, triglyceride glucose-waist circumference (WC) index (TyG-WC = TyG index × WC (cm)) had the highest predictability in females, closely followed by lipid accumulation product (LAP), a novel lipid combined anthropometric index used as a surrogate of visceral obesity and insulin resistance, derived from a gender-specific formula by merging WC and TG [120]. In the young population, HbA1c was found to be a reliable discriminator for people at risk of developing diabetes [121]. Low levels of sRAGE also seem to be a good indicator for the risk of diabetes, cardiovascular disease, and death in the general population [122].

## 8. Phenotypes at Risk for Cardiovascular Complications

The data regarding which diagnostic parameter better predicts the progression to diabetes and the risk for ASCVD are conflicting. One recent study states that HbA1c performed best in forecasting which patients would remain prediabetic or become diabetic. On the other hand, patients who were classified as prediabetic through IGT criteria and regressed to normoglycemia had a reduction in ASCVD risk and death [123].

Which age group is the most affected? Is there an age group where the prevalence is higher? Increased adiposity, low-grade inflammation, and mitochondrial dysfunction in elderly patients might make them ideal candidates [124,125]. However, current studies report they are not the high-risk category we are looking for [107].

Identification of high-risk prediabetic sub-phenotypes might lead to early recognition of patients prone to progression to diabetes and ASCVD. To this end, a very recent study uses measurements linked to the glucose metabolism as well as variables of diabetes pathogenesis in order to sub-classify prediabetes. These variables are HDL, IR, insulin secretion, IFG, a genetic risk score for T2DM risk, and MRI measurements of fat compartments and liver fat content [31]. In order to simplify and to perform phenotyping where the data above were not available for another cohort, they used theoretically similar variables such as glycemia during glucose challenge, insulin sensitivity, insulin secretion, fasting insulin, fasting triglycerides, waist circumference, hip circumference, BMI, and HDL cholesterol. The authors classified intermediate hyperglycemic status into sub-phenotypes pertaining to six different clusters. Only clusters 3 and 5 had a high risk of diabetes and cardiovascular disease. Cluster 3 has low insulin secretion and high genetic risk, and cluster 5 has a high liver fat content and high IR. Both also had a risk of nephropathy, but only sub-phenotype 5 had a high mortality risk. Another interesting finding was that there was a category of patients who had an increased risk of developing renal disease and all-cause mortality independent of the development of T2DM [31,36].

**Figure 2:**
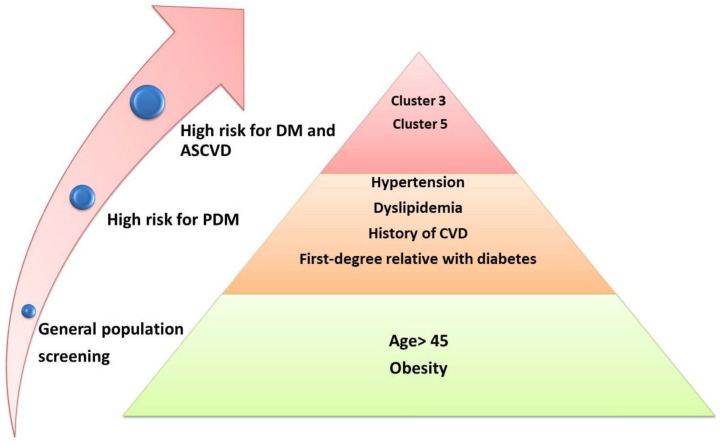
A stratified screening process for prediabetes. ASCVD, atherosclerotic cardiovascular disease; cluster 3, high-risk prediabetic phenotype with low insulin secretion and high genetic risk; cluster 5, high-risk prediabetic phenotype, high liver fat content, and high IR [31]; CVD, cardiovascular disease; DM, diabetes mellitus; PDM, prediabetes.

Phenotyping can go as far as the financial possibilities of different health systems allow it to go. There can be large-scale screening programs for classical risk factors, with a yearly follow-up, or there can be more complex screening as long as it is economically feasible. For large-scale screening, we must decide which test best fits the need to be inexpensive, accurate, and a good predictor for future progression to diabetes and cardiovascular events. Most guidelines recommend that we use a combination of two tests. Maybe an initial, gross screening process could identify patients susceptible to complications, and patients with certain characteristics could be further investigated (see Figure 2).

## 9. When Should We Act? Prevention

Which of the already diagnosed prediabetic patients should undergo closer monitoring and primary prevention interventions? It seems that lowering the thresholds for defining prediabetes increases the number of patients, but dilutes the number of patients at risk of developing diabetes and cardiovascular disease [22]. Indeed, the cut-off values for defining prediabetes might be established depending on the end-game: prevention, targeted therapeutical intervention, risk stratification, or prediction.

There is a greater proven benefit of primary prevention in averting the progression to diabetes and the reduction of macrovascular complications than there is for secondary prevention [29]. The best predictors of glycemic progression to diabetes were analyzed in several prospective studies (See Table 3) [126].

**Figure 3:**
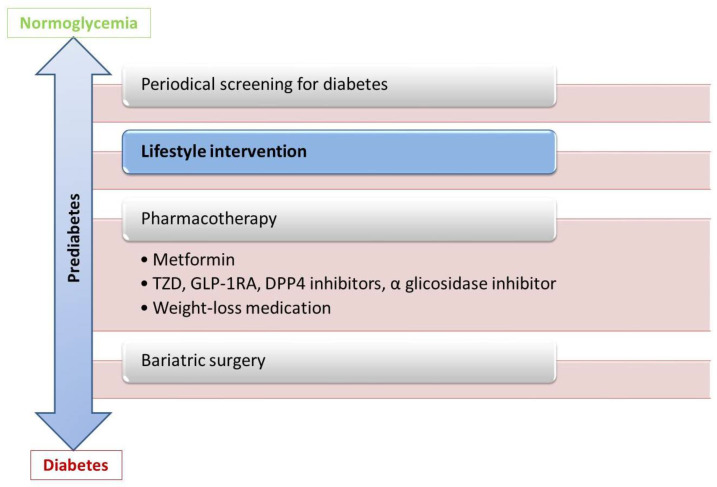
Possible interventions in intermediate glucose states. DPP4—Dipeptidyl peptidase-4, GLP—1RA-Glucagon-like peptide-1 receptor agonist, TZD—thiazolidinediones.

Patients in the early stage of developing T2DM may benefit from personalized diagnosis and therapeutical strategies (see Figure 3). Lifestyle modification is the most efficient approach [61,127]. Pharmacological interventions are reserved for patients who do not respond enough to weight loss, increased physical exercise, or dietary changes. Individuals with IFG might benefit from prevention strategies that primarily target the maintenance of beta-cell mass, while patients with IGT could benefit from treatment that increases peripheral insulin sensitivity. Metformin and Pioglitazone, drugs with minimal effect on insulin secretion, have less effect on non-obese individuals. In contrast, obese patients with prediabetes treated with Metformin registered a delay in the development of T2DM [34]. There is an ongoing clinical trial that investigates the effect of lifestyle and pharmacological intervention on the microvascular function in prediabetic patients, given the fact that prediabetes was also associated with diabetic nephro/neuro/retinopathy [128]. Based on the evidence of subclinical myocardial damage produced by prediabetes (see Section 6.1, a recent article hypothesizes that sodium-glucose cotransporter-2 (SGLT-2) inhibitors administered in the prediabetic state might play a role in the prevention of heart failure (mainly HFpEF) in patients with diabetes mellitus [129]. Bariatric surgery has an already proven benefit on other cardiovascular risk factors, such as hypertension or dyslipidemia, but also leads to normoglycemia in prediabetic and even diabetic patients [130].

## 10. Conclusions

The current definitions of prediabetes are similarly accurate in predicting the risk of progression to diabetes but differ in matters of cardiovascular disease forecasting. Thus, the need for a universal, fit-for-purpose definition of prediabetes remains, with lower cut-offs for prevention and higher cut-offs for risk stratification and prognosis. High-risk prediabetic patients develop ASCVD complications prior to developing T2D, as proven by subclinical myocardial damage and high prevalence of prediabetes among acute ASCVD cases. Thus, early intervention to minimize risk in these patients underscores the need for timely identification by phenotyping of prediabetics. Routine screening for prediabetes should be performed in all patients with classical risk factors for atherosclerotic disease or in those where the macrovascular or microvascular complications are already present. Patients in the higher risk groups may benefit from personalized diagnostic and therapeutic strategies.

## Figures and Tables

**Table 1:** Definitions of prediabetes [6,7].

Definition	Criteria	Prediabetes Range
ADA	IFG	100–125 mg/dL (5.6–6.9 mmol/L)
	IGT	140–199 mg/dL (7.8–11.0 mmol/L)
	HbA1c	5.7–6.4% (39–47 mmol/mol)
WHO	IFG	110–125 mg/dL (6.1–6.9 mmol/L)
	IGT	140–199 mg/dL (7.8–11.0 mmol/L)
IEC	HbA1c	6–6.4% (42–47 mmol/mol)

IFG—impaired fasting glucose, IGT—impaired glucose tolerance, expressed as 2 h post load glucose level, ADA—American Diabetes Association, IEC—International Expert Committee, WHO—World Health Organization.

**Table 2:** Diabetes risk factors checklist for screening [67].

Physical inactivity First-degree relative with diabetes High-risk race/ethnicity Hypertension (≥140/90 mmHg or medicated hypertension) HDL-C < 35 mg/dL or triglycerides > 250 mg/dL Other disorders accompanied by insulin resistance: severe obesity, acanthosis nigricans, polycystic ovaries syndrome Gestational diabetes History of CVD

**Table 3:** Predictors of progression to diabetes [126].

Predictor	Association to Diabetes Progression
IFG	Exponential progression in the top quartile
IGT	Linear increase in progression to diabetes
HbA1c	Good predictor for the young population
Race	Hispanic, Mexican-Americans, Pima, Nauruan populations
High BMI	Good predictor in low-risk populations
Weight gain	Progression to diabetes in African Americans

BMI—body mass index, IFG—impaired fasting glucose, IGT—impaired glucose tolerance.
